# Spontaneous Aching Pain and Peculiar Involuntary Movements: A Case Report of Painful Legs and Moving Toes and Review of the Literature

**DOI:** 10.1155/2014/581402

**Published:** 2014-11-03

**Authors:** Yang-yi Fan, Yan Xu, Xu-guang Gao

**Affiliations:** Department of Neurology, Peking University People's Hospital, Beijing, China

## Abstract

Painful legs and moving toes (PLMT) is a rare syndrome characterized by spontaneous neuropathic pain and peculiar involuntary movements in the lower limbs, especially the toes and feet. As it is a relatively rare disorder worldwide, the exact pathophysiology still remains a mystery. Until recently, numerous methods of clinical treatments have been tried; however, the success rate of the therapies is still very low. Here, we report a case of PLMT and also summarize the recent clinical and research literatures regarding clinical presentation, electrophysiological features, etiology, treatment methods, and prognosis of this disorder. Doctors should be aware of this rare syndrome in a patient with painful and/or restless legs. On the other hand, multiple clinical treatments should be tried, even those which usually produce a poor outcome.

## 1. Introduction

The painful legs and moving toes (PLMT) is a syndrome which was first described by Spillane et al. in 1971 [1]. Clinically, it is characterized by spontaneous aching pain in the lower limb and involuntary movements in the affected toes or feet [1]. Since this syndrome is relatively rare, most of the descriptions of PLMT have been limited to case reports and the incidence remains unknown. The etiology is varied, which includes a series of clinical disorders. The pathophysiologic origin of this disease is still unclear. Numerous treatment approaches, such as oral medications, local nerve blocks, and injection of botulinum toxin type A, have been attempted; however, the therapeutic effects in most patients reported were usually temporary and not ideal [2]. In this report, we present one patient with PLMT syndrome in our department and also make a summary of literatures about this disorder accordingly.

## 2. Case Report

A 29-year-old female was referred to our department with the chief complaints of aching pain and involuntary movements in her feet. Her right ankle was sprained two years ago (July 2011), resulting in the persistent swelling and pain in the injured region. Later, this burning pain with 8/10 intensity in her right ankle and foot was spread to the right leg, which has been worsened in the cold weather or after walking while being relieved in the warm temperature or in the condition of resting. Six months later (January 2012), the onset of involuntary flexion-extension movements of all her right toes was observed at rest. The movements were intermittent in the conscious period and disappeared in sleep. Moreover, in July 2013, the patient described tearing pain that occurred in her left ankle while walking, along with involuntarily abnormal movements presented in her left toes which were similar to those in her right toes. Walking was difficult for her due to the pain. As the symptoms could not be alleviated after resting in bed for four to five weeks, the patient was referred to our hospital.

The patient was generally healthy in the past and there was no history of psychiatric illness or use of neuroleptics. Our clinical examination found arrhythmic flexion-extension movements of bilateral toes repetitively in the resting condition, which could be reduced when touching the dorsum of the foot (see the Supplemental Video in Supplementary Materials available online at http://dx.doi.org/10.1155/2014/581402). The neurological exam was normal. Additional tests including routine and biochemical studies of blood, urine, and stool, thyroid function, serum ceruloplasmin, serum iron level, serum vitamin B12, antinuclear antibody, SSA, SSB, and serum protein electrophoresis showed results that were physiologically normal. CT scan of the head, MRI of the spine, EEG, EMG, and nerve conduction studies did not display pathological finding. MRI of the right ankle revealed soft tissue swelling and joint effusion in that region. The patient was treated by oral baclofen (10 mg three times daily) and gabapentin (300 mg three times daily) for one week. As no significant improvement was obtained, the patient discontinued the treatment. A telephone follow-up for the patient was conducted once every three months. After discharge from our hospital, she accepted the treatment of traditional Chinese medicine and the acupuncture, and also no improvement was observed. We performed the latest follow-up in September 2014, the pain and involuntary movements in her feet still persisted, and the patient attempted to take the botulinum toxin type A injection in Peking Union Medical College Hospital.

## 3. Discussion

A search was undertaken in the PubMed and CNKI databases, by using the keyword “painful legs and moving toes,” to review titles and abstracts for literatures published from 1971 to 2013. A total of 29 relevant articles were finally reviewed.

### 3.1. Clinical Features

Painful legs and moving toes (PLMT) is a rare syndrome which was first reported in 1971 [1]. To date, the incidence and prevalence of PLMT remain unclear since only more than 70 cases have been documented worldwide [2, 3], including 5 cases in China. Currently, the understanding of PLMT is mainly from these case reports. For the incidence, Hassan and his colleagues investigated 76 cases of PLMT and revealed that the incidence in female (66%) tends to be higher than that in male [2]. The mean age of onset in the largest case series was 58 years old, with a range of 24–86 years [2]. The youngest patient reported was 11 years old [4]. Most cases were sporadic, with only one report about a mother and her daughter who both presented with this syndrome [5].

PLMT is characterized by pain in the foot or calf and involuntary movements of the toes, which can be developed unilaterally or bilaterally. Pain is noted in 95% of patients and usually precedes the onset of abnormal movements by days to years. The pain usually continuously occurred in the foot or leg with tingling, tearing, shooting, or burning sensation, which brings more suffering to patients compared to the annoyance of the involuntary movements [2, 3]. However, there is a minority of patients who have little painful feeling but only show discomfort described as formication, tightness, heaviness, or icy coldness. Although the pain in PLMT patients seems to be neuropathic, it does not radiate along the nerve roots and dermatome [4]. Unlike the restless leg syndrome, the pain in PLMT could not be relieved by walking or exercise [4].

Most commonly, the involuntary movements occur in the distal parts of the lower limb. In the literature, toe movements appear in all patients, which consist of flexion/extension, adduction/abduction, clawing/straightening, and fanning/circular movements. The proximal muscles of the limb and even the thigh muscles in individuals could be affected when the involuntary movements are severe. The movements are usually continuous, or intermittent in some cases, varying in different patients but tending to be stereotypical to a particular individual [2]. Moreover, the movements in PLMT patients are difficult for ordinary people to imitate, even by the patient himself. Some of the patients can stop the movements for a short time by conscious effort while others suffer more severe movements when concentrating on the affected toes. As most involuntary movements, the movements in PLMT tend to be more obvious in tension or pain but disappear in sleep. Conversely, it is stated in a few reports that the toe movements in patients persist or even become more severe in sleep [6]. Generally, the involuntary movements are ipsilaterally associated with the pain, while being contralaterally associated with the pain in a few individuals reported [7].

In a small number of the patients, the clinical presentation of PLMT, pain and involuntary movements, has involved other parts of the body, leading to the development of some PLMT variants including painful arm and moving fingers (PAMF), painful hands and moving fingers (PHMF), painful limbs/moving extremities (PLME), and a few cases without pain, which is called painless limbs/moving extremities [8]. Typically, the movements are accompanied by the pain and disappear when the pain was relieved. But in some cases, the involuntary movements were not limited as the pain has been relieved. Conversely, in the individuals treated with botulinum toxin, their movement symptoms were reduced while the pain persisted [2].

### 3.2. Electrophysiological Observations

The abnormality of the somatosensory evoked potential (SEP) has been found in some patients, specifically showing the prolonged latency and decreased conduction, which indicate the pathological damage in the peripheral to central sensory pathway. The velocity of the peripheral nerve conduction could be normal and the indications of peripheral neuropathy or radiculopathy could be presented as well. In addition, EMG examination is able to reveal two types of activities: one is the random, unpredictable, irregular bursts which can last 80–1000 ms and the other is the semicontinuous bursts with a similar duration. The latter is also associated with cocontraction of antagonistic and contiguous muscles of which the frequency is 1.5–200 Hz [2, 9].

### 3.3. Etiology and Pathogenesis

The cause of PLMT syndrome is still not fully understood. In addition to a few patients with idiopathic onset [9], its possible etiologies reported include peripheral nerve lesion, neuropathy (polyneuropathy from diabetes, alcoholism, tarsal tunnel syndrome, lumbar spinal stenosis, or hypertrophic mononeuritis), vitamin B12 deficiency, Sjögren syndrome, systemic lupus erythematosus, IgG monoclonal gammopathy, herpes zoster myelitis, spinal cord compression, Hashimoto's disease, Wilson disease, human immunodeficiency virus, and neuroleptics [9]. In cases reported in China, one was idiopathic [10], and three patients had Wilson's disease [7]. Peripheral neuropathy and local trauma account for the most common cause [2, 3], such as our case that was demonstrated above in which right foot injury induced onset of PLMT.

There are still controversies regarding the origin and pathophysiological mechanism of the pain and the movements. The two retrospective studies conducted by Hassan et al. and by Liu et al. demonstrate that both neuropathy and radiculopathy are the most common causes of PLMT [2, 3]. The nature of the pain is usually described as tingling, tearing, shooting, or burning, which is the typical characteristic of the neuropathic pain. Patients with radiculopathy triggered by local foraminal stenosis [8] have symptoms relieved after surgical decompression, suggesting that the potential lesion may originate in the peripheral nervous system. On the other hand, there is no evidence of peripheral damage in some patients combined with Wilson's disease [11] or Hashimoto's disease [12]. Moreover, in patients with local trauma or unilateral peripheral neuropathy, their pain and involuntary movements emerge bilaterally [2]. Further, the blockade of peripheral nerve or nerve root is not able to relieve the symptoms completely and substantially [2]. These findings indicate that the origins of lesions might involve not only peripheral nerve, but also central nervous system. Specifically, it is speculated that neural discharges formed in the peripheral lesion site activate ventral horn cells in the spinal cord to produce the toes movements [13]. The lesions in peripheral nerve or nerve root may lead to changes in function of the neural afferent inputs to the upstream neurons in the brainstem and subcortical centers, resulting in a pathological process in the neural reorganization and integration, which further initiate an imbalance of excitatory/inhibitory signals to the downstream neurons [14]. Different degrees in the alteration of sensory afferent function and the central reorganization may be the key determinants of the variety of clinical manifestations in PLMT [15]. According to these, it is proposed by Alvarez et al. [9] that the involuntary movements are most likely originated from the spinal cord or higher center (e.g., basal ganglia) while the pain may be derived from the central or peripheral nerves. Peripheral neuropathy and soft tissue trauma are assumed to be precipitating factors of the pathological changes in the sensory function and the central reorganization. In addition, in two PLMT cases reported by Drummond and Finch [16], the pain and involuntary movements could be triggered by the factors such as coldness and startle, which can increase the sympathetic activity. It is also found that both pain and movements are relieved after the inhibition of sympathetic system. Nonetheless, such relief is temporal, only lasts for a few days, and is followed by the reoccurrence of the pain and involuntary movements. This implies that the sympathetic activity may also contribute to the cause of the symptoms in PLMT. Sympathetic neural discharge seems to be a facilitator of nociceptive impulses in the affected limbs. The pain and involuntary movements of the toes finally appeared in both sides in our patient and there was no evidence of peripheral neuropathy or radiculopathy in clinical physical examination or EMG studies, which suggests that a central origin may be more plausible in this patient. Because the EMG was undertaken over two years later after the trauma in the right ankle, the possibility of associated subclinical transient neuropathy should be considered. Although the exact pathophysiological mechanism could not be identified, we propose that functional changes of the neural afferent inputs initiated by the soft tissue damage and the subsequent central neuron reorganization in the spinal cord or the higher centers might play a major role in the pathogenesis of our patient.

### 3.4. Treatment and Prognosis

Currently, there is no evidence that PLMT syndrome can be spontaneously resolved over time. Although a vast array of drugs has been used for the treatment, the success rate of the therapies is still limited. The drugs include baclofen, benzodiazepines, antidepressants, antiepileptics, NSAIDs, and levodopa. The GABAergic agents, such as gabapentin and pregabalin, are known to inhibit calcium influx through voltage-gated calcium channels in the dorsal root ganglia and spinal cord and, therefore, interrupt the series of events that lead to neuropathic pain. They also can centrally inhibit GABA in the basal ganglia to suppress centrally generated movements. The GABAergic agents are found to be effective in attenuating the pain and the movements in PLMT patients, possibly via both central and peripheral mechanisms [9, 17, 18]. In the study conducted by Alvarez et al., gabapentin or pregabalin offered positive effect in half of the 14 cases. The maximum dose used for gabapentin is 2400 mg daily, and for pregabalin it is 1500 mg daily [9]. Surgical decompression may be effective in patients with radiculopathy caused by foraminal stenosis or spinal stenosis [8, 19]. Local nerve blockade, epidural anesthesia, sympathetic blockade, or epidural spinal cord stimulation can relieve the pain and also reduce the movements. However, their therapeutic effects are usually temporary; repeated treatments are required to achieve sustained curative effects [8, 16, 20, 21]. Local botulinum A injection under electromyographic guidance was reported to be helpful in pain relief and movement control, but the symptoms return after a few days or months [10, 22, 23]. After repeated injections, complete remission of pain and movements could be sustained for more than 3 years [23]. The botulinum A is suggested to act via a reduction of muscle spindle discharge leading to a decrease of activity of the gamma loop and central sensitization.

Although a list of therapeutic options is available, it is worth noting that many patients still had poor responses to the therapies, and a few had symptoms aggravated gradually along with the progression of the disease. Among all these patients, there are no unique characteristics in the aspects of their sex, age of onset, nature of pain, and neurologic examination [2].

## 4. Conclusion

PLMT is a rare syndrome. Until recently, only more than 70 cases have been reported worldwide [2], including 5 cases which were found in China. We reported one patient with characteristic pain and involuntary movements of PLMT. The etiology and exact mechanism still remain unknown, and the evidence of possible etiologies reported has not been found in our patient, indicating a more complex pathophysiological mechanism of this syndrome. Baclofen and gabapentin were not helpful in our patient. Additionally, we also first reported the treatments using traditional Chinese medicine and acupuncture for this syndrome, and unfortunately poor effect was observed.

## Supplementary Material

The involuntary flexion-extension movements presented in the right toes of the PLMT patient, which could be reduced when touching the ankle or the dorsum of the foot.
